# Survivin, a key player in cancer progression, increases in obesity and protects adipose tissue stem cells from apoptosis

**DOI:** 10.1038/cddis.2017.209

**Published:** 2017-05-18

**Authors:** Miriam Ejarque, Victòria Ceperuelo-Mallafré, Carolina Serena, Gisela Pachón, Yaiza Núñez-Álvarez, Margarida Terrón-Puig, Enrique Calvo, Catalina Núñez-Roa, Wilfredo Oliva-Olivera, Francisco J Tinahones, Miguel Angel Peinado, Joan Vendrell, Sonia Fernández-Veledo

**Affiliations:** 1 Hospital Universitari de Tarragona Joan XXIII, Institut d´Investigació Sanitària Pere Virgili Universitat Rovira i Virgili, Tarragona, Spain; 2 CIBER de Diabetes y Enfermedades Metabólicas Asociadas, Instituto de Salud Carlos III, Madrid, Spain; 3 Department of Dermatology, Program of Excellence in Glycosciences, Brigham & Women’s Hospital/Harvard Medical School, Boston, MA, USA; 4 Department of Medicine, Program of Excellence in Glycosciences, Brigham & Women’s Hospital/Harvard Medical School, Boston, MA, USA; 5 Health Sciences Research Institute Germans Trias i Pujol, Institute of Predictive and Personalized Medicine of Cancer, Badalona, Spain; 6 CIBER de la Fisiopatología de la Obesidad y la Nutrición, Instituto de Salud Carlos III, Madrid, Spain; 7 Laboratory of Biomedical Research, Virgen de la Victoria Clinical University Hospital, Málaga, Spain

## Abstract

Adipose tissue (AT) has a central role in obesity-related metabolic imbalance through the dysregulated production of cytokines and adipokines. In addition to its known risk for cardiovascular disease and diabetes, obesity is also a major risk for cancer. We investigated the impact of obesity for the expression of survivin, an antiapoptotic protein upregulated by adipokines and a diagnostic biomarker of tumor onset and recurrence. In a cross-sectional study of 111 subjects classified by body mass index, circulating levels of survivin and gene expression in subcutaneous AT were significantly higher in obese patients and positively correlated with leptin. Within AT, survivin was primarily detected in human adipocyte-derived stem cells (hASCs), the adipocyte precursors that determine AT expansion. Remarkably, survivin expression was significantly higher in hASCs isolated from obese patients that from lean controls and was increased by proinflammatory M1 macrophage soluble factors including IL-1*β*. Analysis of survivin expression in hASCs revealed a complex regulation including epigenetic modifications and protein stability. Surprisingly, obese hASCs showed survivin promoter hypermethylation that correlated with a significant decrease in its mRNA levels. Nonetheless, a lower level of mir-203, which inhibits survivin protein translation, and higher protein stability, was found in obese hASCs compared with their lean counterparts. We discovered that survivin levels determine the susceptibility of hASCs to apoptotic stimuli (including leptin and hypoxia). Accordingly, hASCs from an obese setting were protected from apoptosis. Collectively, these data shed new light on the molecular mechanisms governing AT expansion in obesity through promotion of hASCs that are resistant to apoptosis, and point to survivin as a potential new molecular player in the communication between AT and tumor cells. Thus, inhibition of apoptosis targeting survivin might represent an effective strategy for both obesity and cancer therapy.

Obesity has reached epidemic proportions in Western cultures, and its comorbidities, particularly diabetes and cardiovascular diseases, are major public health concerns.^1^ In response to chronic positive energy balance, white adipose tissue (WAT) expands by increasing the volume of pre-existing adipocytes (hypertrophy) and by generating new adipocytes (hyperplasia) through the recruitment of adipocyte progenitor cells termed adipose-derived stem cells (ASCs).^2^ Current nonsurgical options for obesity are largely unsuccessful over the long term because once hyperplasia occurs the new adipose cells remain even after weight loss.^3, 4^ Energy restriction is associated with a decrease in adipocyte cell size but not in number; thus, the irreversibility of hyperplasia might explain the ‘yoyo’ effect of easy regain of lost weight.^5^ Consequently, promotion of adipose tissue (AT) apoptosis to reduce total fat mass has recently emerged as a promising therapeutic strategy for the treatment of obesity.^6^

Intriguingly, epidemiological studies confirm an association between obesity and several forms of cancer.^7^ Proposed mechanisms that link obesity/adiposity to high cancer risk include obesity-related inflammation and/or adipokine production via enhancement of cell proliferation and migration, and inflammation pathways, which can prompt cancer development and metastasis.^8^ Apoptosis or anti-apoptosis (cell survival) mechanisms are usually at the crossroads of the main pathways influenced by dysfunctional adipokine production in obesity. Local pro-tumorigenic effects of ASCs have been also highlighted as important in this process, with a dual role in the dynamics of WAT expansion and in tumor progression by inducing proliferation and survival of some cancer cells through the release of angiogeneic and proinflammatory factors.^9, 10^ In this context, we have recently shown that the clinical phenotype exerts a profound influence on the behavior of ASCs, with enhanced proliferation but impaired adipogenic differentiation in ASCs isolated from obese subjects,^11^ underscoring the notion that obesity is linked to defective adipocyte turnover. However, the apoptotic sensitivity of ASCs in an obese context is unknown.

Survivin is a member of the inhibitor of apoptosis family of proteins and functions to inhibit apoptosis and promote cell division.^12^ Survivin expression is developmentally regulated in normal tissue, with little expression in most terminally differentiated tissues. The abnormally high expression of survivin in cancer cells makes it an attractive anticancer target. However, accumulating evidence indicates potential functions for survivin in normal tissues, indicating that survivin expression is not cancer specific.^13^ Interestingly, survivin expression is upregulated by adipokines such as leptin, suggesting that the molecular effects of adiposity on carcinogenesis might be mediated by suppression of apoptosis through survivin-dependent mechanisms.^14, 15^ Moreover, it has been demonstrated that survivin is regulated by cytokines in lymphocytes and plays an important role in proliferation and survival of hematopoietic cells.^16^

To our knowledge, no studies have examined the impact of obesity on survivin expression. We hypothesized that an obese environment could promote survival of the ASC niche by decreasing its sensitivity to apoptosis via a survivin-dependent mechanism. We show for the first time that circulating levels and WAT expression of survivin are increased in human obesity. Furthermore, we demonstrate that survivin protein expression is elevated in ASCs isolated from obese individuals, and protects from apoptosis. These findings outline a regulatory for survivin as a new player in AT remodeling with potential impact in the mechanisms linking obesity/adiposity to increased cancer risk.

## Results

### Circulating levels and WAT expression of survivin are elevated in obesity

**Figure 1:**
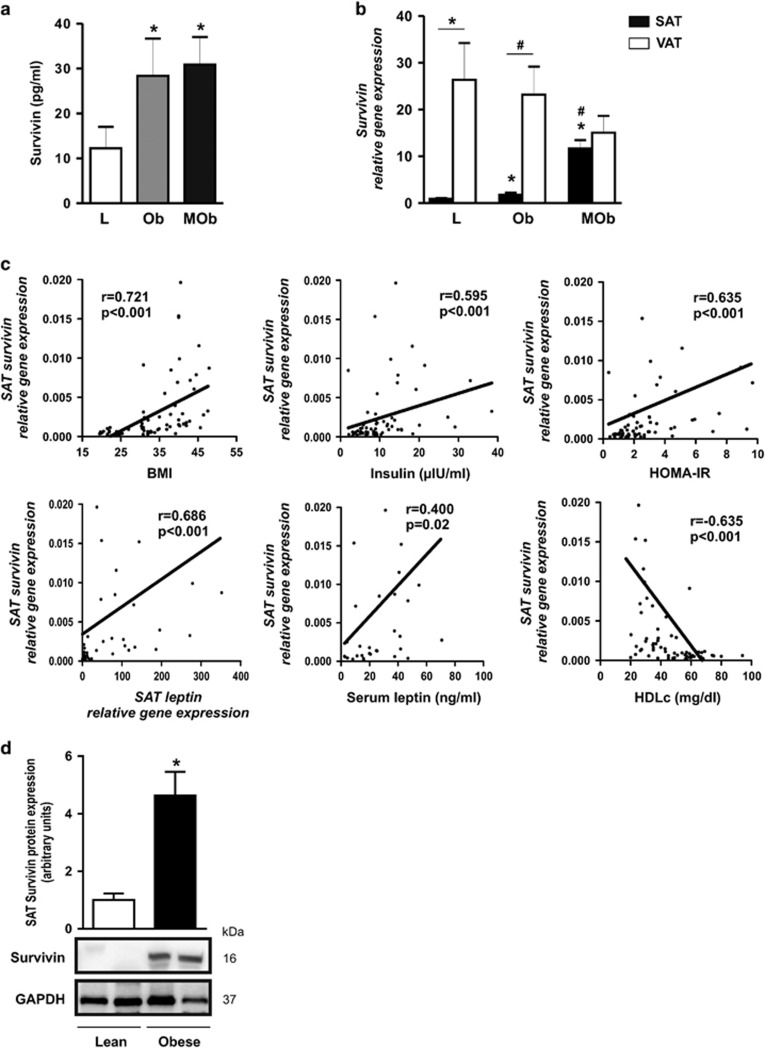
Obesity increases circulating survivin levels and gene expression in SAT. (**a**) Survivin levels in serum of subjects classified according to BMI (L=lean, BMI<25; Ob=obese, 25<BMI>35; MOb=morbid obese, BMI>35) (*n*=32 L, 41 Ob and 38 MOb). *P<0.05 *versus* lean. (**b**) Survivin mRNA expression in SAT and VAT depots from lean, obese and morbid obese subjects (*n*=32 L, 41 Ob and 38 MOb). *P<0.01 *versus* lean and ^#^*P*<0.01 *versus* obese. (**c**) Correlation between SAT survivin mRNA expression and BMI, insulin, HOMA-IR, SAT leptin gene expression, serum leptin and HDLc. (**d**) SAT survivin protein levels in obese and lean subjects. *P<0.01 (*n*=8 lean and 8 obese). Data information: All values are expressed as mean value±S.E.M. Statistical analyses: For (**a**and **b**), ANOVA with *post hoc* Scheffe correction; for (**c**), Spearman’s correlation analysis, and for (**d**), unpaired Student’s test

We first determined whether obesity impacted the circulating levels of survivin by measuring its concentration in subjects according to body mass index (BMI) (clinical and laboratory data summarized in Table 1). Circulating survivin levels were significantly higher in obese and morbid obese subjects than in lean subjects (Figure 1a), and positively correlated with BMI in bivariate analysis (*r*=0.274; *P*=0.031). A positive association was also found between serum survivin and both circulating levels of leptin (*r*=0.3487; *P*=0.009) and leptin gene expression in subcutaneous AT (SAT) (*r*=0.471; *P*=0.011), which is consistent with studies linking leptin and survivin function in some tumors.^14^ We next measured survivin gene expression in SAT and visceral AT (VAT) depots from the same subjects. In both lean and obese subjects, survivin expression was significantly higher in VAT than in SAT (Figure 1b). We also found a significant increase in survivin mRNA expression in SAT across the groups, with morbid obese subjects having the highest expression (Figure 1b), whereas no differences in survivin mRNA expression were observed in VAT across the different groups. Bivariate analysis showed that survivin gene expression in SAT correlated positively with BMI, insulin, HOMA-IR, SAT leptin mRNA and leptin serum levels (Figure 1c) and negatively with HDLc (*r*=−0.634; *P*<0.001). To strengthen the independence of these associations as predictors of survivin gene expression, a multiple regression analysis model was constructed including the above-mentioned bivariate correlations, adjusting for age and gender. SAT survivin expression was predicted by BMI and HDLc (*b*=0.036 and *β*=0.008, *P*<0.001; *b*=−0.008 and *β*=0.004, *P*=0.056, respectively; Supplementary Table S1). To validate the finding of increased survivin gene expression in SAT from obese subjects, we measured survivin protein levels by immunoblotting. In accordance with changes in gene expression, survivin protein expression in SAT was significantly higher in obese subjects than in lean subjects (Figure 1d). Collectively, our results demonstrate for the first time that both circulating concentrations and SAT expression levels of survivin are increased in human obesity.

### Survivin is predominantly expressed in hASCS of obese subjects

**Figure 2:**
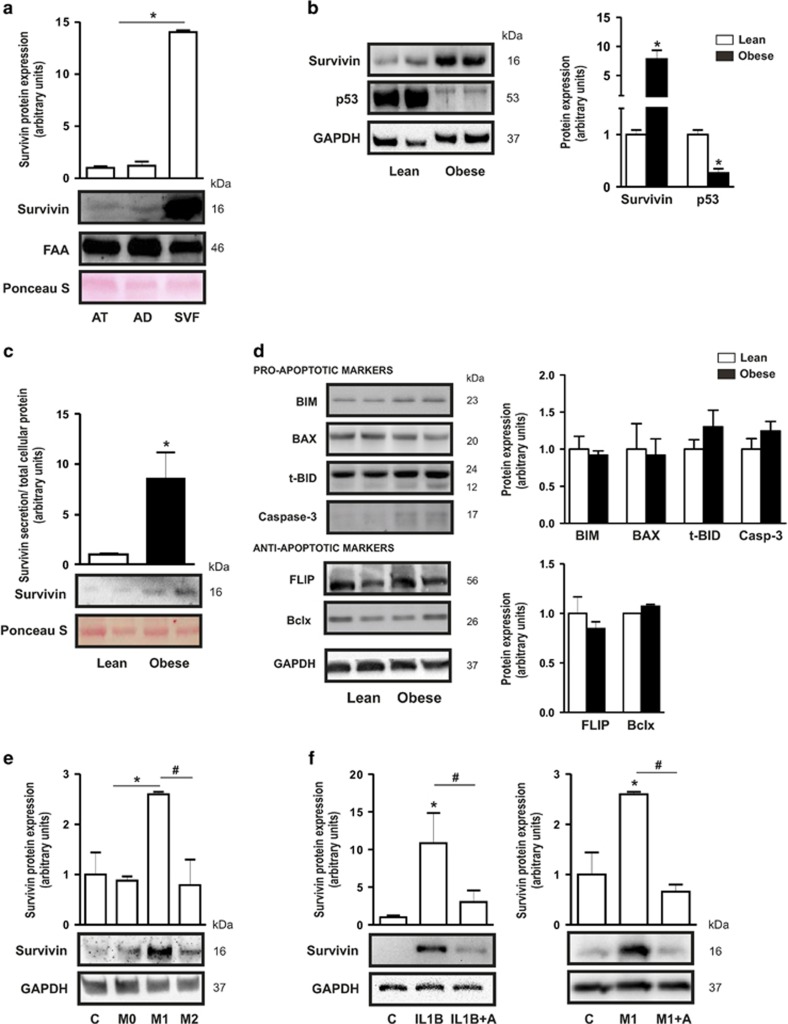
Survivin is predominantly expressed in obese hASCs and is influenced by the inflammatory environment. (**a**) Survivin protein expression and quantification in total adipose tissue (AT), adipocyte fraction (AD) and stromal-vascular fraction (SVF) from SAT of lean and obese patients. Fumarylacetoacetase (FAA) was used as a loading control. Ponceau S staining is also provided. *P<0.001 *versus* total AT and AD. (**b**) Survivin and p53 protein levels and quantification in hASCs from lean and obese patients. GAPDH was used as a loading control. *P<0.01 *versus* lean. (**c**) Survivin secreted levels and quantification in medium from lean- and obese-derived hASCs. Ponceau S staining was used to check loading. *P<0.05 *versus* lean conditioned medium. (**d**) Total cell extracts of lean- and obese-derived hASCs were subjected to immunoblotting with antibodies against apoptotic and antiapoptotic markers and GAPDH was used as a loading control. No significant differences were found. (**e)** Lean hASCs were cultured in RPMI medium (C: control) or for 24 h in conditioned medium (CM) from macrophages (M0, M1 and M2) and cells were subjected to immunobloting with survivin and GAPDH antibodies. *P<0.05 *versus* CM from control, and M0; ^#^*P*<0.05 *versus* M2. (**f**) Left panel, lean hASCs were cultured in RPMI medium (C: control) or RPMI supplemented with IL-1*β* (IL1B) or IL-1 *β* and IL-1 *β* receptor antagonist (**a**); right panel, with CM of M1 macrophages or CM of M1 and IL-1*β* receptor antagonist (**a**). Survivin protein levels were measured and GAPDH was used as a loading control. Quantification of three experiments is shown. *P<0.05 *versus* control (**c**); ^#^*P*<0.05 as indicated in figure. Data information: Values are expressed as mean±S.E.M. Statistical analysis: unpaired Student’s *t*-test. *n*=3–4 patients for each group

Given that alterations in survivin levels occurred only in SAT, we focused our attention on this compartment. To identify survivin-expressing cells within the SAT depot, we extracted the adipocyte and stromal-vascular fraction (SVF) from SAT and measured survivin protein expression. Immunoblotting analysis of cell lysates showed that survivin was chiefly expressed in the SVF, with barely detectable levels in adipocytes (Figure 2a). The stroma surrounding mature adipocytes is known to contain a heterergeneous population of cells rich in hASCs, representing approximately 20% of the SVF. We therefore evaluated survivin protein expression in subcutaneous hASCs isolated from lean and obese subjects. Survivin expression was significantly higher in obese- than in lean-isolated hASCs (Figure 2b). Consistent with the described survivin-p53 regulatory axis,^17^ high levels of survivin protein in obese-isolated hASCs were accompanied by significantly lower levels of p53 (Figure 2b). Furthermore, significantly higher amounts of survivin were detected in conditioned medium (CM) from cultured obese-isolated hASCs than in equivalent CM from lean-isolated hASCs, suggesting greater secretion (Figure 2c). A modest but nonsignificant increase in the expression of t-BID and caspase-3 proteins was also detected in obese hASCs (Figure 2d, top panel), similar to that described in obese WAT.^18, 19^ No differences were detected in the expression of other pro-apoptotic (Figure 2d, top panel) or antiapoptotic (Figure 2d, bottom panel) markers.

We next questioned whether the local proinflammatory environment characteristic of AT in the obese state might underlie survivin protein overexpression in obese hASCs. Thus, we explored the effect of secreted factors of both proinflammatory M1 and anti-inflammatory M2 macrophages for survivin expression. To do this, THP-1 cells were polarized to an M1 or an M2 phenotype as described in Materials and Methods and CM was collected and used to culture lean-derived ASCs for 24 h. Survivin expression was significantly higher in hASCs cultured in CM from proinflammatory M1 than in CM from M2 or M0 macrophages (Figure 2e), suggesting that survivin could be regulated by M1 macrophage soluble factors. Indeed, treatment of lean-derived hASCs with the proinflammatory cytokine IL-1*β* was sufficient to enhance survivin expression, which was blocked by the IL-1*β* receptor antagonist (IL-1RA) (Figure 2f, left panel). Consistent with a role for IL-1*β* in survivin upregulation, M1-induced survivin expression in lean hASCs was blunted in cells co-treated with IL-1RA (Figure 2f, right panel). Taken together, these results suggest that macrophage–ASC crosstalk in obese AT controls the homeostasis and properties of adipocyte precursors, at least for apoptosis susceptibility.

### Obesity regulates survivin expression in hASCs by epigenetic, post-transcriptional and post-translational mechanisms

**Figure 3:**
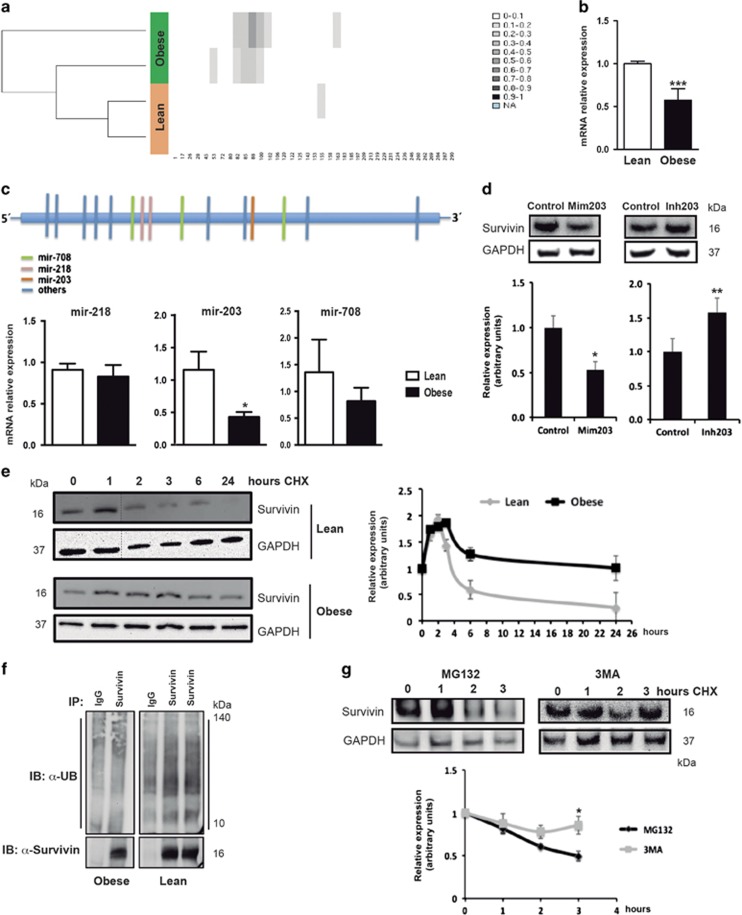
Survivin is regulated at several levels. (**a**) Hierarchical clustering analysis of DNA methylation levels shows higher ratios of methylation on the survivin promoter from obese-derived hASCs. (**b**) Survivin mRNA expression in hASCs from lean and obese donors. ***P<0.001 *versus* lean. (**c**) Survivin expression regulated by specific miRNAs binding to the 3′-UTR of survivin mRNA. *P<0.05 *versus* lean. (**d**) hASCs were treated with a mimic (mim203) or inhibitor (inh203) of miRNA-203 and cell lysates were subjected to immunoblotting against survivin and GAPDH. A representative blot and densitometry analysis of three independent experiments performed are shown. *P<0.05; **P<0.01 *versus* control. (**e**) hASCs were treated with 50 *μ*g/ml cycloheximide (CHX). At the indicated time, cells were lysed and equal amounts of protein were subjected to immunoblotting with anti-survivin and anti-GAPDH antibodies. A representative blot and the densitometry analysis of three independent experiments performed are shown. (**f**) hASCs cells from lean and obese patients were lysed and immunoprecipitated with an anti-survivin antibody or IgG control. Immunoprecipitates were analyzed by immunoblot using anti-ubiquitin antibody (anti-Ub). Survivin was used as a loading control. Image is representative of three independent experiments. (**g**) Lean hASCs were pre-treated with MG132 or 3-MA before cycloheximide (CHX) treatment. At the indicated hours of CHX incubation, cells were lysed and equal amounts of protein were subjected to immunoblotting with anti-survivin and anti-GAPDH antibodies. A representative blot and the densitometry analysis of three independent experiments performed are shown. *P<0.05 *versus* MG132. Data information: Values are expressed as mean±S.E.M. Statistical analysis: Student’s test. *n*=3–4 patients for each group

Notwithstanding the finding that IL-1*β* secreted by M1 macrophages might be at least partly responsible for the increased levels of survivin in obese-derived hASCs, the fact that levels were maintained during routine culture of hASCs (Figure 2b), when the inflammatory signal is no longer present, suggested changes in the epigenome, which are relatively persistent. We hypothesized that obese-derived hASCs are obesity conditioned by epigenetic modifications; specifically, that obesity-linked hypomethylation of the survivin promoter was responsible for its overexpression in obese hASCs. Accordingly, we analyzed the methylation status of the 5´untranslated region of exon 1 of the survivin gene in hASCs isolated from lean and obese subjects. Contrary to our expectation, methylation-specific PCR and pyrosequencing revealed that the survivin promoter was hypermethylated in obese-derived hASCs (Figure 3a). A similar association has been detected in endometrial tumors where DNA methylation is related to inhibition of p53-mediated repression of survivin.^20^ Nevertheless, we found that survivin promoter hypermethylation in obese-derived hASCs was accompanied by a significant decrease in the levels of its mRNA (Figure 3b), indicating that additional regulatory mechanisms should exist to explain the high levels of survivin protein in hASCs from obese subjects. Given the discrepancy between mRNA (Figure 3b) and protein expression (Figure 2b), we sought to analyze potential differences in the expression of the miRNAs miR-218, mi-R203 and miR-708, which are among the best known miRNAs to target survivin.^21^ We observed that whereas the expression levels of miR-218 and miR-708 were unchanged between lean- and obese-derived hASCs, the level of miR-203 was significantly decreased in obese-derived hASCs (Figure 3c). Considering that miRNAs bind to the 3´UTR of survivin mRNA and alter protein translation, the finding of reduced miR-203 expression correlates well with enhanced survivin protein expression and is consistent with a number of studies in cancer cell lines.^22^ To confirm that survivin is a direct target of miR-203 in our cellular system, we analyzed the effect of miR-203 on survivin protein expression in SGBS cells, a well-established human preadipocyte cell line.^23^ Survivin protein expression was significantly reduced in cells transfected with an miR-203 mimic, whereas an miR-203 inhibitor significantly increased survivin protein levels (Figure 3d). Because regulation of survivin expression is complex and also occurs at post-translational levels,^24^ we also explored protein stability. Using cycloheximide (CHX) to block *de novo* protein synthesis, we measured the steady-state levels of survivin protein in obese- and lean-derived hASCs. Immunoblotting analysis revealed altered kinetics of protein stability depending on the donor tissue, with greater survivin protein stability in obese-derived hASCs (Figure 3E). As previously observed for other proteins,^26, 27, 28^ CHX treatment provoked an early increase in steady-state levels of survivin both in obese and lean hASCs; the origin of this is unknown. Survivin can be degraded via the ubiquitin–proteasome pathway and is turned over rapidly with a half-life of about 30 min.^25^ Levels of ubiquitinated-survivin were higher in lean hASCs than in obese hASCs (Figure 3f), suggesting that an obese environment regulates the steady-state levels of survivin through the proteasomal degradation pathway. Unexpectedly, treatment of lean-derived hASCs with MG132, an inhibitor of the ubiquitin–proteasome system, failed to block degradation of survivin (Figure 3g). By contrast, the autophagy inhibitor 3-methyladenine (3-MA) stabilized survivin protein, pointing to a potentially important role for the autophagy-dependent degradation pathway for survivin protein stability. Other protein-ubiquitin conjugates have likewise been described as target proteins for proteasome-independent degradative pathways.^29^ Overall, our results demonstrate that obesity determines survivin protein levels in hASCs via several mechanisms that would be most likely controlled by an epigenetic signature associated with the obese environment.

### Survivin levels determine apoptosis sensitivity of hASCs

**Figure 4:**
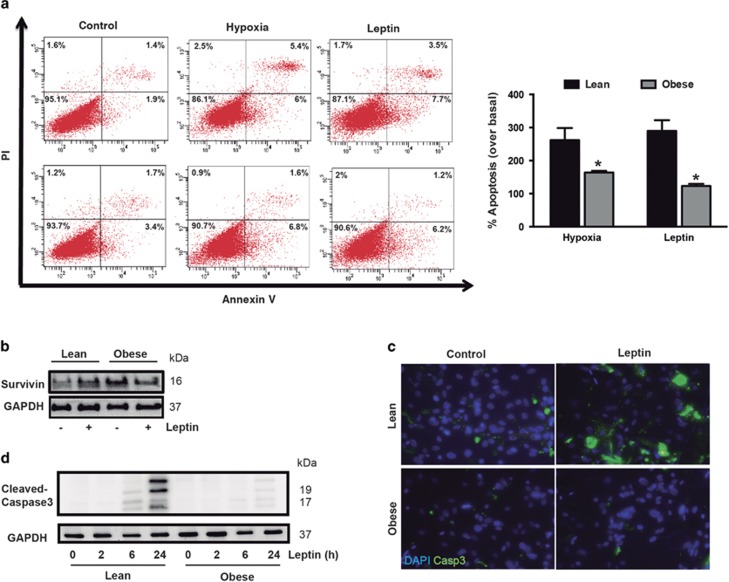
Obese hASCs are resistant to apoptotic stimuli. (**a**) Flow cytometry analysis of annexin V+propidium iodide (PI) staining in hASCs from lean and obese subjects treated with different apoptotic stimuli for 48 h. Left panel, representative dot-blots of the different conditions analyzed. Right panel, quantification of % apoptosis over basal (untreated cells) by annexinV staining. Data were normalized to their controls. *P<0.05 *versus* lean. (**b**) Survivin protein levels in leptin-treated hASCs from lean and obese patients. GAPDH was used as a loading control. (**c**) Immunofluorescence staining for cleaved caspase-3 (green) and DAPI (blue) in hASCs. (**d**) Total cell extracts of lean- and obese-derived hASCs were subjected to immunoblotting with anti-cleaved caspase-3, showing increased caspase-3 levels in lean hASCs treated with leptin for 48 h. Data information: Values are expressed as mean±S.E.M. Statistical analysis: Student’s *t*-test. *n*=3 for each group

**Figure 5:**
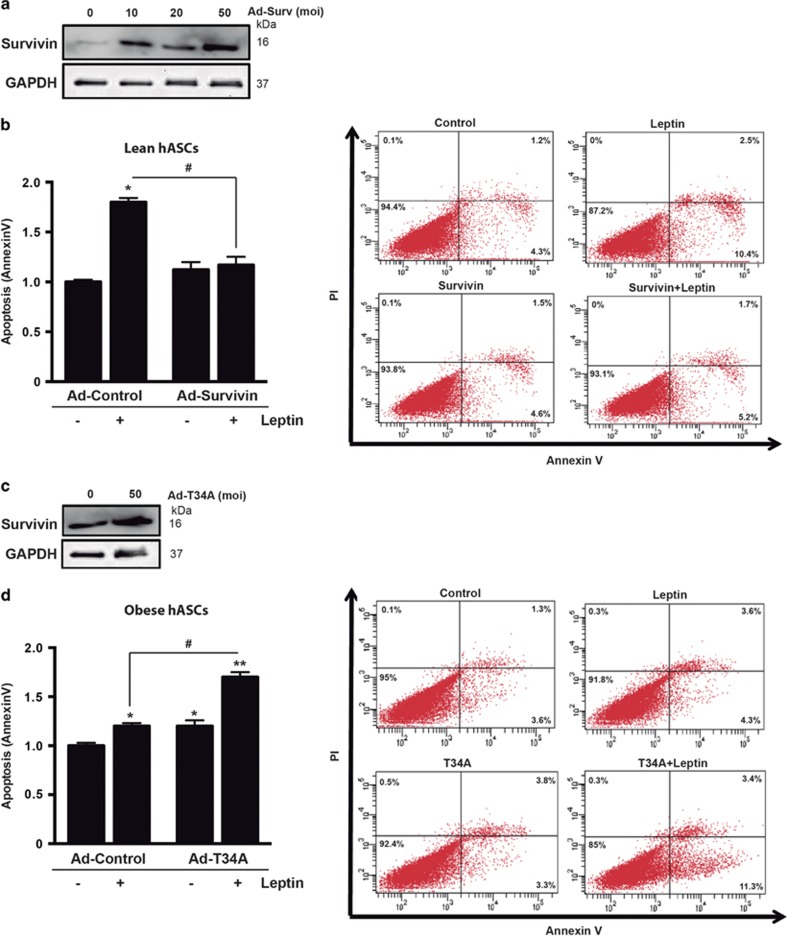
Survivin levels determine apoptotic sensitivity of hASCS. (**a**) Lean hASCS were infected with recombinant adenovirus expressing HA-tagged survivin (Ad-Surv). Different titrations of the recombinant survivin adenovirus were assessed to establish the best concentration to be used. A representative immunoblot is shown. (**b**) Lean hASCs were infected with Ad-control or Ad-survivin and 16 h after infection, cells were incubated with or without leptin for 48 h followed by annexin V+propidium iodide (PI) staining and flow cytometry. Left panel, quantification of annexinV staining. *P<0.01 *versus* control; ^#^*P*<0.05 *versus* Leptin. Right panel, representative dot-blots of the different conditions analyzed. (**c**) Obese hASCS were infected with a recombinant adenovirus expressing a mutated survivin protein (Ad-T34A). A representative immunoblot is shown. (**d**) Obese hASCs were infected with Ad-control or Ad-T34A and 16 h after infection, cells were incubated with or without leptin for 48 h followed by annexin V+propidium iodide (PI) staining and flow cytometry. Left panel, quantification of annexinV staining. *P<0.01, **P<0.001 *versus* control; ^#^*P*<0.001 *versus* Leptin. Right panel, representative dot-blots of the different conditions analyzed. Data information: Values are expressed as mean±S.E.M. Statistical analysis: Student’s *t*-test. *n*=3 patients for each group

Similar to other adult stem cell niches, self-renewal and apoptosis of hASCs represent major processes that determine *in vivo* maintenance and AT mass.^30^ Our previous studies revealed that the microenvironmental niche triggers intrinsic alterations in the biology of hASCs that affect their proliferation and differentiation;^11, 31, 32^ however, their apoptotic susceptibility has not been studied to date. Given that survivin functions as an anti-apoptosis protein, we evaluated the sensitivity of hASCs to apoptosis induced by hypoxia and leptin, two recognized obesity-related apoptotic stimuli in AT.^33, 34, 35^ As anticipated, basal apoptosis was not significantly different between obese- and lean-isolated hASCs cells (Figure 4a), as previously reported.^30^ Both hypoxia and leptin significantly increased the number of apoptotic cells in lean- but not in obese-derived hASCs, as detected by double staining with annexin V and propidium iodide (Figure 4a). At the protein level, leptin increased survivin levels in lean but not in obese hASCs, where the levels were already high under basal conditions (Figure 4b). Additionally, the level of caspase-3 cleavage, as evaluated by immunocytochemistry (Figure 4c) and immunoblotting (Figure 4d), was also higher in leptin-treated lean-isolated hASCs than in equivalent obese-isolated cells. Thus, our results not only provide the first evidence that leptin induces apoptosis of hASCs, which differs from data on murine 3T3-L1 preadipocytes,^36^ but also reveal a general protection from apoptosis in obese hASCs. Finally, we explored whether survivin expression was linked to the suppression of apoptosis in hASCs. We first treated hASCs isolated from obese donors with YM155, a small-molecule transcriptional repressor of survivin,^37^ and we evaluated apoptosis in cells treated with leptin. Surprisingly, YM155 treatment failed to sensitize obese hASCs to the pro-apoptotic effects of leptin (Supplementary Figure 1A), most likely because YM155 did not decrease survivin protein expression, whereas mRNA levels were progressively reduced in a dose-dependent manner (Supplementary Figure 1B). Nevertheless, adenovirus-mediated overexpression of survivin (Figure 5a) resulted in the suppression of apoptosis in leptin-treated lean-derived hASCs (Figure 5b), mimicking the phenotype of obese-derived hASCs. Conversely, adenovirus-mediated overexpression of dominant-negative survivin (T34A mutation) (Figure 5c) in an obese context increased the sensitivity to leptin-induced apoptosis, essentially reverting the obese phenotype (Figure 5d).

Overall, our data demonstrate that survivin regulates sensitivity to apoptosis of hASCs. Because of its differential expression in hASCS from lean and obese subjects, survivin might represent a promising drug target for inhibition of AT expansion.

## Discussion

It has been proposed that the inability of AT to adequately expand triggers adipocyte apoptosis, a key initial event that contributes to macrophage infiltration and the chronic low-grade inflammatory state characteristic of obesity, in turn creating a pro-apoptotic milieu in AT of obese patients. However, it is evident that fat mass expansion in WAT is not prevented in obese patients^2^ and, in fact, the increase in cell numbers characteristic of hyperplastic obesity contributes to the enlargement of fat stores in advanced obesity.^38^ We hypothesized that the obese environment promotes survival of the adipocyte precursors not only by increasing their proliferation rate^11^ but also by decreasing their sensitivity to apoptosis. Specifically, we demonstrate that hASCs are involved in the apoptotic resistance of AT in obesity, in part via epigenetic regulation of survivin expression, a key regulator of apoptosis with an important role in tumor expansion. Furthermore, we provide evidence that levels of local and systemic survivin are regulated in obesity, and we identify a close relationship between survivin and leptin, an adipokine associated with a damaging inflammatory and antiapoptotic environment that favors tumor growth and cancer progression.^39^

It was the prevailing view that blockade of apoptotic cell death would reduce AT inflammation and the metabolic consequences of obesity.^40^ However, induction of massive adipocyte-specific apoptosis induces the infiltration of alternatively activated M2 macrophages, but not of proinflammatory M1 macrophages, which are characteristic of obese AT.^41^ Along this line, recent studies reveal that increasing the rate of programmed adipocyte death reduces the number of adipocytes and prevents diet-induced obesity and further weight gain under a positive energy balance.^6^ Collectively, these results suggest that not only mature adipocytes but also their precursors are critical in this equilibrium. In this regard, we have recently shown that an obese environment increases the proliferative potential of hASCs,^11^ which might encourage adipocyte turnover through promotion of the hASC niche. In the present work, we have discovered a new mechanism that may go some way to help understand the events that take place in AT in the context of obesity, namely survivin-mediated hASC resistance to apoptotic cell death in obese subjects. Thus, obesity increases the adipocyte progenitor pool by increasing cell proliferation and inhibiting apoptosis. Although one might think that this would be an ideal scenario for promoting SAT expansion, which has been proposed as a viable therapeutic strategy to improve glucose homeostasis and insulin resistance,^42^ the obese environment in this depot is also associated with restricted adipogenesis.^11, 43^ Therefore, we propose that in an obesogenic context, the maintenance of SAT homeostasis is altered as a consequence of intrinsic defects in hASCs.

Many of the characteristics attributed to hASCs from an obese phenotype reproduce the behavior of tumor cells: robust proliferation,^11^ apoptotic resistance (this study) and an invasive phenotype.^32^ Interestingly, the antiapoptotic effects of some adipokines have been highlighted as a mechanism of cell survival promoting cancer progression. Recent findings have shown a strong relationship between adipocyte-derived leptin and breast cancer.^44^ Notwithstanding the fact that leptin might act centrally to trigger apoptosis in WAT resulting in adipocyte depletion,^45, 46, 47^ several studies suggest that the molecular effects of leptin on carcinogenesis are mediated by suppression of apoptosis through survivin-dependent mechanisms.^14, 15^ Chronically high levels of leptin in obesity can lead to leptin resistance,^48^ which results not only in loss of appetite control but also in resistance to adipocyte apoptosis. Importantly, we show here that circulating survivin levels are increased in obesity, and are positively correlated with circulating leptin. Survivin has been implicated both in suppression of cell death and regulation of mitosis and is found in several subcellular locations such as mitochondria, cytoplasm, nucleus and the extracellular space.^49^ Higher circulating levels of survivin have been detected in patients with prostate and pancreatic cancer^50, 51^ and in patients with rheumatoid arthritis.^52^ The source of extracellular survivin is an intriguing but unsolved question; however, the increased expression observed in SAT from obese patients leads us to speculate about an adipose origin. Specifically, our study reveals that survivin is predominantly expressed in hASCs, which concurs with previous studies that define a role for survivin in regulating the function of hematopoietic progenitor cells.^16, 53, 54^ Our data clearly establish that the levels of survivin in hASCs determine the susceptibility of these cells to apoptotic stimuli.

Survivin has been proposed as a key node protein involved in adaptation to unfavorable environments since it assists in protecting cells from stresses such as hypoxia, imbalanced pH, and altered metabolic and redox states commonly found in the microenvironment of tumors.^55^ Therefore, it is not unreasonable to assume a function for survivin in hASCs, especially in hASCs from an obese environment. Extracellular survivin might be significant for the pathogenesis of inflammation as previously reported in human leukocytes in the context of autoimmune arthritis^56^ or asthmatic pregnancy.^57^ In its extracellular context, survivin modulates T-cell responses, skewing immunity towards a Th2 response^58^ similar to the phenotype observed in obese patients where leptin resistance is developed.^59^ Moreover, downregulation of survivin results in a decrease of IL-6 production by human monocytes.^60^ Overall, an additional function of survivin in modulation of inflammatory state in obesity should not be ruled out.

Nonetheless the proinflammatory microenvironment of AT in obesity may be sufficient to explain the increased levels of survivin in hASCs. In fact, the higher levels of survivin in VAT compared with SAT depots, particularly in lean subjects, might be the result of the basal inflammatory state characteristic of visceral depots where an increased expression of proinflammatory cytokines such as IL-1*β* was detected (Supplementary Figure S2). However, the maintenance of these levels *ex vivo* points to obesity-induced epigenetic changes of the survivin gene. Our data also show that the obese microenvironment in SAT determines survivin protein levels in hASCs via several mechanisms including post-transcriptional and post-translational control of gene expression, as previously reported in cancer cell lines.^21^ Although several strategies have been developed to target survivin in cancer therapy,^61^ our study in hASCs suggests that simply turning off the survivin transcriptional machinery will not be sufficient to decrease survivin levels and increase the apoptotic sensitivity of obese hASCs. The differential expression of survivin in obese- and lean-isolated hASCs is a strong rationale for development of survivin-based obesity therapeutics targeting the ASC niche.

Although further studies will be required to fully understand the role of survivin in AT, our findings open a new perspective on AT dynamics, where survivin might act as a nodal protein controlling apoptosis and inflammation. They may also help to unravel the regulatory pathway governing crosstalk between AT and tumor cells. Thus, inhibition of apoptosis targeting survivin might represent an effective strategy both for obesity and cancer therapy.

## Materials and methods

### Study selection and sample processing

One hundred and eleven subjects were recruited by the Endocrinology and Surgery departments at the University Hospital Joan XXIII (Tarragona, Spain). All subjects were of Caucasian origin and reported that their body weight had been stable for at least 3 months before the study. They had no systemic disease other than obesity, and all had been free of infection in the previous month before the study. Primary liver disease, cardiovascular disease, arthritis, acute inflammatory disease, infectious disease, neoplasic and renal diseases were specifically excluded by biochemical work-up. Subjects were classified by BMI according to World Health Organization criteria as lean, obese and morbid obese. Subjects were stratified according to age, gender and BMI. The hospital ethics committee approved the study and informed consent for biobanking surgically removed tissue was obtained from all participants in accordance with the Declaration of Helsinki. All patients had fasted overnight before collection of AT and blood. VAT and SAT were obtained from the same individual during scheduled non-acute surgical procedures including laparoscopic surgery for hiatus hernia repair or cholecystectomies in non-morbid obese population. Samples from morbid obese subjects were obtained during bariatric surgery. AT samples were washed in PBS and immediately frozen in liquid N_2_ with storage at −80 °C, or used immediately for fractionation. For AT fractionation, fresh AT was diced into small pieces (10–30 mg), washed in PBS and incubated in Medium 199 (Gibco, Gran Island, NY, USA) plus 4% BSA and 2 mg/ml collagenase type I (Sigma-Aldrich, St. Louis, MO, USA) for 1 h in a shaking water bath at 37 °C. Adipocytes were separated by filtration through a 200-*μ*m mesh fabric (Spectrum Laboratories, Rancho Domínguez, CA, USA) and centrifugation for 5 min at 1500 × *g*. Buoyant adipocytes were carefully removed from the top layer and the pellet consisted of stromal-vascular cells.

### Blood analysis

Blood samples were drawn after a 12-h fast, before surgery. Serum was separated and immediately frozen at –80 °C. Serum biochemical parameters were measured in duplicate. Serum glucose, cholesterol, HDL cholesterol and triglycerides were measured by standard enzymatic methods (Randox Laboratories Ltd, Antrim, UK). Insulin was measured with an immunoradiometric assay (BioSource International, Camarillo, CA, USA). The HOMA-IR was calculated from fasting insulin and glucose using the following equation: HOMA-IR=fasting insulin (*μ*IU/ml) × fasting glucose (mol/l)/22.5. Serum survivin was measured by a sandwich ELISA (R&D Systems, Minneapolis, MN, USA). The assay sensitivity was 1.58–9.96 pg/ml and the intra- and inter-assay co-efficients of variance (CVs) were less than 5.5 and 9.5%, respectively.^62^ Serum leptin was measured by ELISA (R&D Systems). The minimum detectable concentration of human leptin is typically less than 7.8 pg/ml and the intra- and inter-assay CVs were less than 3.3 and 5.4%, respectively.

### Cell culture and treatments

hASCs were isolated from AT of lean and obese patients from a subsample of patients (donor information summarized in Table 2) following published protocols. After isolation, the minimal functional and quantitative criteria established by the International Society of Cell Therapy (ISCT) and the International Federation for Adipose Therapeutics and Science (IFATS) were routinely confirmed by flow cytometry as described (Supplementary Figure S3).^32^ All experiments were performed in cells at passages 3–7. Cells were cultured in DMEM/F12 medium containing 10% FBS and 1% antibiotic/antimycotic (penicillin, streptomycin and fungizone) at 37 °C in a humidified incubator (21% O_2_ and 5% CO_2_). For hypoxia experiments, cells were cultured in a modular incubator with 2% O_2_, 93% N_2_ and 5% CO_2_ during 48 h. Adenovirus expressing wild type or T34A-mutated survivin^63^ was added to 2 × 10^5^ cells in a 35-mm plate at a multiciplity of infection of 50, followed by incubation for 2 h at 37 °C in Opti-MEM Medium (Gibco). An empty adenovirus was used as a control. After incubation, adenovirus-containing medium was replaced with standard culture medium. Twenty-four hours later, cells were treated with 8 *μ*g/ml leptin (Peprotech, Offenbach, Germany) for 24−48 h to induce apoptosis. To inhibit survivin expression, obese-derived hASCs were cultured with 20 nM of YM155 (Calbiochem, La Jolla, CA, USA) a potent survivin inhibitor,^37^ prior to leptin treatment. Since YM155 is highly toxic and might trigger cell death, dose and timing was empirically determined by mRNA analysis of survivin. The human acute monocyte leukemia cell line THP-1 was obtained from the ATCC (Rockville, MD, USA) and cultured as described.^64^ Briefly, to obtain macrophages, cells were cultured in RPMI-1640 medium supplemented with 10% FBS, 1% antibiotic/antimycotic and 0.1 *μ*g/ml phorbol myristate acetate (PMA; Sigma) in a humidified incubator at 37 °C with 5% CO_2_ for 72 h. Adherent cells (M0 macrophages) were then cultured in the same medium but without PMA for 24 h. M0 macrophages were polarized to M1 or M2 macrophages with 250 ng/ml LPS (Fluka, Buchs, Switzerland) or 50 ng/ml IL-4 (Peprotech), respectively, overnight (Supplementary Figure S4). Conditioned medium was collected after 48 h.

### miRNA mimic and inhibition assays

miR-203a-3p mimic or miR-203a-3p hairpin inhibitor (50 nM) (GE Dharmacon, Chicago, IL, USA) or their respective controls were transfected into SGBS cells using Lipofectamine 3000 (Invitrogen, Carlsbad, CA, USA). Cells were incubated at 37 °C, 5% CO_2_ for 48 h and harvested for analysis.

### Gene expression analysis

Total RNA was extracted from AT/cells using the RNeasy Lipid Tissue Midi Kit (Qiagen, Hilden, Germany). miRNA and RNAs <200 nucleotides were isolated from hASCs using the miRNeasy Mini kit (Qiagen). Total RNA quantity was measured at 260 nm and purity was assessed by the OD_260_/OD_280_ ratio. For gene expression analysis, 1 *μ*g of RNA was reverse transcribed with random primers using the Reverse Transcription System (Applied Byosistems, Foster City, CA, USA). For miRNA analysis, cDNA synthesis was performed with the TaqMan MicroRNA Reverse Transcription Kit (ThermoFisher Scientific, Waltham, MA, USA). Real-time PCR (qPCR) was conducted on a 7900HT Fast Real-Time PCR System using TaqMan Gene Expression Assays (Applied Biosystems) for survivin (Hs 00426651_m1), leptin (Hs00174497_m1) and specific Taqman probes for hsa-miR-203, hsa-miR-218 and hsa-miR-708. Results were calculated using the comparative Ct method (2-ΔΔCt) and expressed relative to the expression of the housekeeping genes cyclophilin 1A (*PPIA*) (Hs 04194521_s1), *18 S* (Hs 03928985_g1) and *RNU48* (for miRNA analysis).

### Protein expression analysis

Cells were lysed in RIPA buffer containing a protease inhibitor cocktail (Sigma-Aldrich) and equal amounts of protein were separated on SDS-PAGE gels, transferred to Immobilon membranes and blocked. Immunoreactive bands were visualized using SuperSignal West Femto chemiluminescent substrate (Pierce, Rockford, IL, USA) and images were captured using the VersaDoc imaging system and Quantity One software (Bio-Rad, Hercules, CA, USA). The following antibodies diluted 1:1000 were used: anti-survivin (ab76424; Abcam, Milton, Cambridge, UK); anti-BIM and anti-Bclx (559685 and 610211, respectively; BD Pharmingen, San Diego, CA, USA); anti-BAX and anti-cleaved caspase-3 (#2772 and #9664, respectively; Cell Signaling Technology, Beverly, MA, USA); anti-tBID and anti-FLIP (AF860 and AF821, respectively; R&D Systems); anti-GAPDH (MA5-15738; Sigma-Aldrich); anti-FAA and anti-Ub (sc-66223 and sc-8017, respectively; Santa Cruz Biotechnology, PaloAlto, CA, USA). Secondary peroxidase-conjugated antibodies diluted 1:2000 were as follows: anti-goat (HAF109; R&D Systems), anti-rabbit and anti-mouse (NA934 and NXA931, respectively; GE Healthcare, Chicago, IL, USA) and anti-chicken (ab131366; Abcam).

### Protein stability

Protein stability assays employing 50 *μ*g/ml cycloheximide (CHX) (Sigma) to inhibit *de novo* protein synthesis were performed as described.^25^ Cell lysates were subjected to SDS-PAGE and immunoblotting with an anti-survivin antibody (Abcam). For proteasome inhibition, 25 *μ*M of MG132 (Calbiochem) was added 1 h before CHX. For autophagy-dependent protein degradation assay, 5 mM 3-MA was added 4 h before CHX.

### Immunoprecipitation assays

Cell lysates containing equal amounts of protein (200–400 *μ*g) were immunoprecipitated with anti-survivin antibody (Abcam) overnight at 4 °C. Immune complexes were precipitated with protein A/G-agarose beads (Sigma-Aldrich) for 4 h at 4 °C. Immunoprecipitates were separated by SDS-PAGE and immunoblotted.

### Immunocytochemistry

hASCs grown on coverslips were fixed with 4% (w/v) paraformaldehyde, rehydrated with 2% (v/v) fish skin gelatin and permeabilized with 0.2% Triton X-100 prior to incubation with 5% (v/v) goat serum. Subsequently, cells were incubated overnight at 4 °C with anti-cleaved caspase-3 antibody (Cell Signaling Technology) in PBS containing 1% goat serum. Coverslips were washed with PBS and incubated for 1 h at room temperature with 1:100 Alexa Fluor 488 (Life Technologies, Carlsbad, CA, USA) followed by mounting with Pro Long Gold Antifade Reagent with 40,6-diamidino-2-phenylindole, DAPI (Invitrogen). Images were acquired on a Leica DM 4000B fluorescence microscope (Leica Microsystems, Wetzlar, Germany) and captured with a Leica DFC 300 FX camera (Leica Microsystems).

### Cell apoptosis flow cytometry assay

Apoptosis was measured in 1 × 10^5^ hASCs (lean- and obese-isolated hASCs) after the specified treatments. Cells were collected by trypsinization, washed with PBS and resuspended in 100 *μ*l 1 × binding buffer. Annexin V staining was accomplished following the product instructions for the FITC Annexin V/Death Cell apoptosis Kit for flow cytometry (Life Technologies) on a FACSAria III flow cytometer (BD Biosciences, San Jose, CA, USA) with BD FACSDiva software.

### Bisulfite conversion and direct sequencing

Bisulfite conversion was performed using 200 ng of DNA with the EZ DNA Methylation-Gold Kit (ZymoResearch, Orange, CA, USA). Converted DNA was eluted in 30 *μ*l. Bisulfite sequencing was performed following Clark´s procedure.^65, 66^ Briefly, for each region of interest, PCR amplification was first performed on 1 *μ*l of bisulfite-treated DNA using conventional PCR. The PCR product was directly used as a template for nested PCR according to each specific primer set. Samples were purified with the JETQuick PCR Spin Kit (Genomed, St. Louis, MO, USA) and sequenced with a specific primer by the sequencing service of GATC Biotech. Briefly, to assess the DNA methylation state of each cytosine, the raw sequencing electropherograms were analyzed with Geospiza FinchTV software (Perkin Elmer, Cleveland, OH, USA). The height of each peak was measured to assess the proportion of each population. For the sake of simplicity, the methylation level was ranked in five different intervals 0–0.2, 0.21–0.4, 0.41–0.6, 0.61–0.8 and 0.81–1, which reflected the DNA methylation state of the particular cytosine.

### Statistical analyses

Statistical analysis was performed with the Statistical Package for the Social Sciences software, version 15 (SPSS). For *in vitro* data, experimental results are presented as mean±S.E.M. from three to four independent experiments performed at least in duplicate. Statistical significance was tested by unpaired Student’s *t*-test or one-way ANOVA followed by the protected least-significant different test. For clinical and anthropometrical variables, normal distributed data are expressed as mean value±S.D., and for variables with no Gaussian distribution values are expressed as median (interquartile range). For analysis of expression variables that do not have a Gaussian distribution, values were analyzed by non-parametrical tests. Differences in clinical variables, laboratory parameters or expression variables between groups were compared by ANOVA with *post hoc* Scheffe correction. Interactions between factors as well as the effects of covariates and covariate interactions with factors were assessed by Spearman’s correlation analysis. Correction for confounding and interacting variables was performed using stepwise multiple linear regression analysis.

## Figures and Tables

**Table 1:** Anthropometric and biochemical variables

	**Lean**	**Obese**	**Morbid obese**
*N*	32	41	38
Sex (male/female)	(15/17)	(16/25)	(24/12)
Age (years)	52 (43–55)	54 (48–57)	51 (44–53)
BMI (kg/m^2^)	23.06 (2.39)	31.53 (1.55)*	40.61 (3.46)^*,**^
Glucose (mg/dl)	90.50 (82.75–98.50)	104 (96–125)*	91.8 (86.4–102)**
Insulin (*μ*IU/ml)	6.09 (3.77–8.03)	9.00 (6.05–12.68)*	14.22 (11.64–19.44)^*,**^
HOMA-IR	1.24 (0.76–1.63)	2.20 (1.59–3.19)*	3.25 (2.46–4.90)^*,**^
Serum leptin (ng/ml)	8.08 (2.8–15.7)	12.63 (7.11–43.05)*	26.10 (15.67–41.70)*
Leptin SAT (arbitrary units)	3.62 (2.06–4.24)	6.77 (4.91–11.27)	84.02 (53.99–124.16)^*,**^

Abbreviations: BMI, body mass index; HOMA-IR, homeostasis model assessment of insulin resistance index Data are presented as mean (S.D.) or median (25th–75th), as appropriate. Differences were analyzed by the unpaired *t*-test (normal distribution) or Mann–Whitney *U-*test (data not-normally distributed). ^*,**^Indicates significant differences between the means of the different groups: * versus lean and ** versus obese *P*<0.05

**Table 2:** hASCs patient information

	**Lean**	**Obese**
*N*	4	4
Sex	F	F
Age, years	39±2.54	31±3.34
BMI, kg/m^2^	22.35±0.77	33.6±1.05*

Abbreviation: BMI, body mass indexData are presented as mean±S.D. Differences were analyzed by the unpaired *t*-test (normal distribution). *Indicates significant difference *versus* lean *P*<0.001
