# Supplementary material for: Survivin, a key player in cancer progression, increases in obesity and protects adipose tissue stem cells from apoptosis
Source: Cell Death Dis. 2017 May 18;8(5):e2802–. doi: 10.1038/cddis.2017.209 (PMC5520726; doi:10.1038/cddis.2017.209)
Supplement: Supplementary Table 1 [file cddis2017209x1.doc]

**Supplementary Table 1.** Clinical and analytical variables associated with SAT survivin expression

| **Log SAT survivin (R=0.777; R^2^=0.585)** | | | | | |
| --- | --- | --- | --- | --- | --- |
|  | **B (unstandardized)** | **SE** | **95% CI** | **Beta (standardized)** | **p** |
| constant | -3.693 | 0.438 | -0.4577- -0.2809 | - | <0.001 |
| BMI | 0.036 | 0.008 | 0.019-0.053 | 0.568 | <0.001 |
| HDLc | -0.008 | 0.004 | -0.017-0.0002 | -0.265 | 0.056 |

Variables included in the model: age, gender, HOMA-IR, serum leptin, SAT leptin and HDLc
