# Supplementary material for: Survivin, a key player in cancer progression, increases in obesity and protects adipose tissue stem cells from apoptosis
Source: Cell Death Dis. 2017 May 18;8(5):e2802–. doi: 10.1038/cddis.2017.209 (PMC5520726; doi:10.1038/cddis.2017.209)
Supplement: Supplementary Figure 1 [file cddis2017209x2.pdf]

Supplementary Figure 1

A

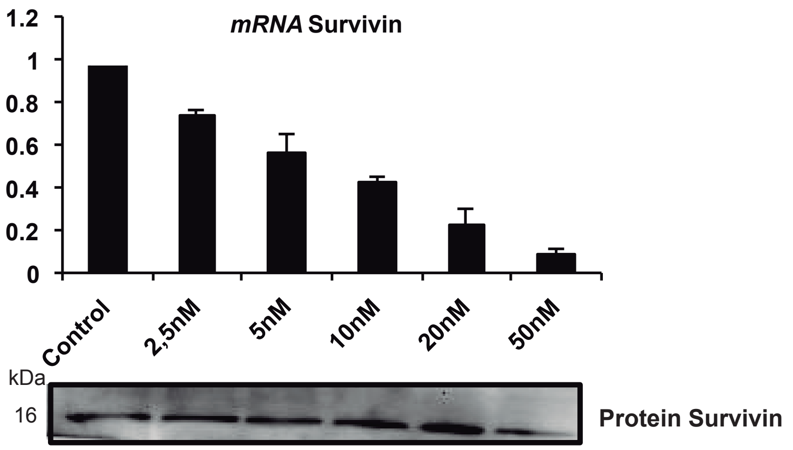

B

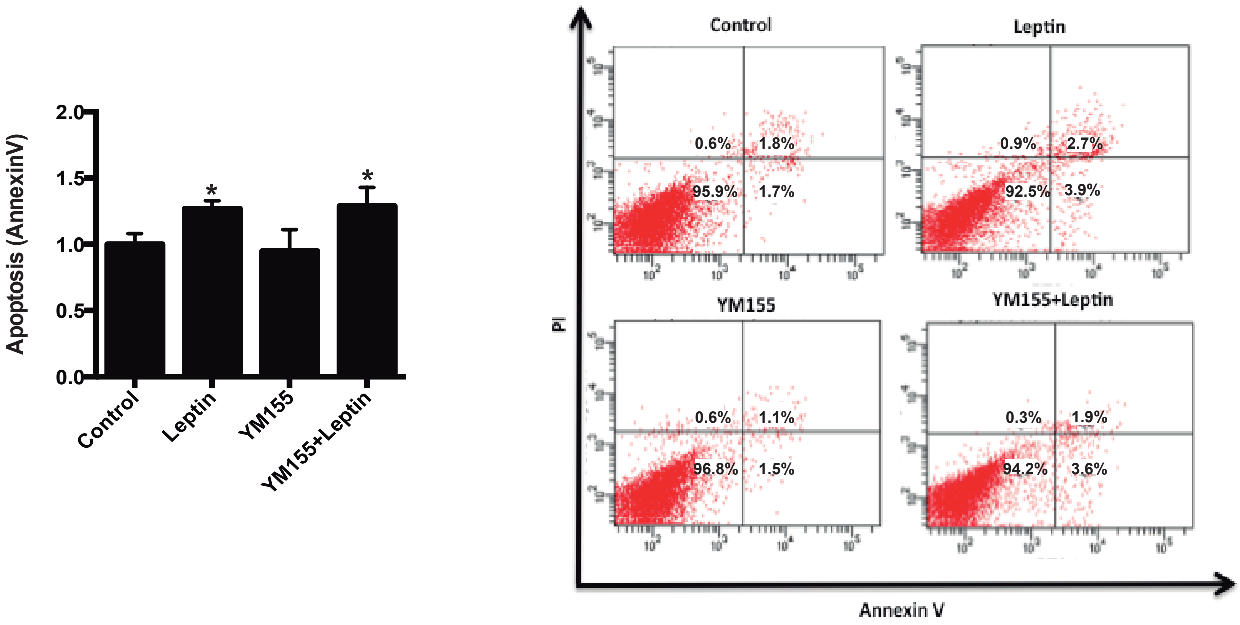

Supplementary Figure 1. YM155 does not decrease survivin protein levels or impact apoptosis.
A) Obese-derived hASCs were treated with different concentrations of the survivin inhibitor YM155 and cells were harvested for mRNA and protein analysis 24h later. YM155 treatment led to a dose-dependent decrease of survivin mRNA but not protein.
B) Obese hASCs were treated with YM155 and the following day they were treated with leptin for 48 h. Left panel, quantification of annexinV staining by flow cytometry. *, $p<0.05$ vs control. Right panel, representative dot-blots of the different conditions analyzed.
Data information: Values are expressed as mean ± SEM. Statistical analysis: Student´s test. n=3 patients for each group.
