# Supplementary material for: Survivin, a key player in cancer progression, increases in obesity and protects adipose tissue stem cells from apoptosis
Source: Cell Death Dis. 2017 May 18;8(5):e2802–. doi: 10.1038/cddis.2017.209 (PMC5520726; doi:10.1038/cddis.2017.209)
Supplement: Supplementary Figure 2 [file cddis2017209x3.pdf]

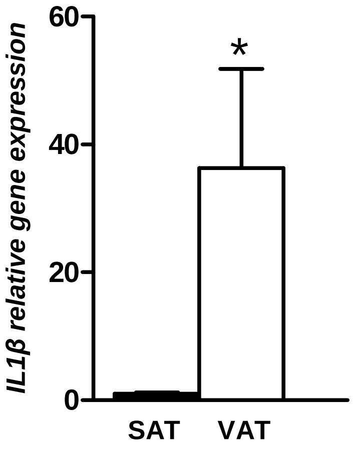

Supplementary Figure 2. VAT had a higher expression of IL1β than SAT. IL1β was analyzed by qPCR in SAT and VAT from lean and obese subjects. Results are mean ± SD from 9 patients. * $P<0.01$ *vs* SAT.
