# Supplementary material for: Survivin, a key player in cancer progression, increases in obesity and protects adipose tissue stem cells from apoptosis
Source: Cell Death Dis. 2017 May 18;8(5):e2802–. doi: 10.1038/cddis.2017.209 (PMC5520726; doi:10.1038/cddis.2017.209)
Supplement: Supplementary Figure 3 [file cddis2017209x4.pdf]

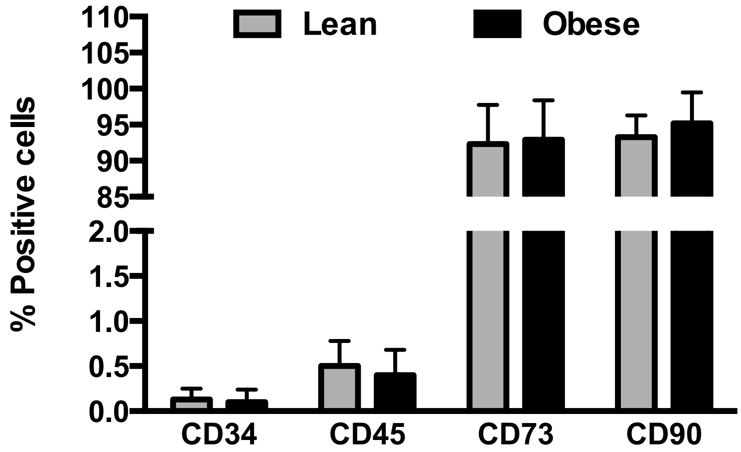

Supplementary Figure 3. Immunophenotypic characterization of hASCs derived from lean and obese individuals. The graph reflects the mean ± SD percent surface positive staining of hASCs for the markers recommended by IFATS (n=4).
