# Supplementary material for: Survivin, a key player in cancer progression, increases in obesity and protects adipose tissue stem cells from apoptosis
Source: Cell Death Dis. 2017 May 18;8(5):e2802–. doi: 10.1038/cddis.2017.209 (PMC5520726; doi:10.1038/cddis.2017.209)
Supplement: Supplementary Figure 4 [file cddis2017209x5.pdf]

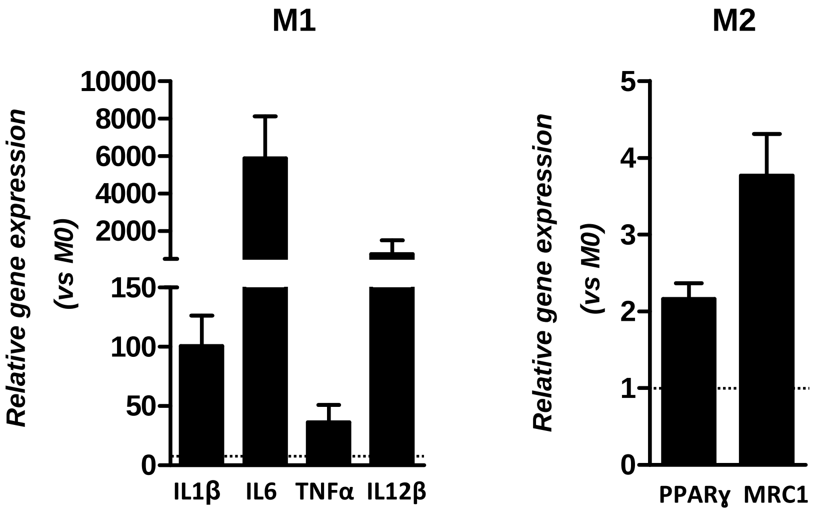

Supplementary Figure 4. M1 and M2 gene expression markers

M0 macrophages were polarized to M1 or M2 macrophages and expression of IL1β IL6, TNFα and IL12β (M1 markers) and PPARɣ and MRC1 (M2 markers) was analyzed by qPCR. Results are mean ± SEM from five independent experiments.
